# LuQi Formula Regulates NLRP3 Inflammasome to Relieve Myocardial-Infarction-Induced Cardiac Remodeling in Mice

**DOI:** 10.1155/2021/5518083

**Published:** 2021-06-28

**Authors:** Xiaoqing Zhang, Dandan Zhao, Jiling Feng, Xiaoli Yang, Zhenzhen Lan, Tao Yang, Xiaoni Kong, Huiyan Qu, Hua Zhou

**Affiliations:** ^1^Department of Cardiovascular Disease, ShuGuang Hospital Affiliated to Shanghai University of Traditional Chinese Medicine, Shanghai, China; ^2^Central Laboratory, ShuGuang Hospital Affiliated to Shanghai University of Traditional Chinese Medicine, Shanghai, China

## Abstract

**Background:**

Excessive activation of the nod-like receptor family pyrin domain containing 3(NLRP3) inflammasome plays a significant role in the progression of cardiac injury. In China, it has been well recognized that Chinese herbal medicine is markedly effective in treating cardiovascular diseases (CVDs). LuQi Formula (LQF) has been used clinically for more than 10 years and confirmed to be effective in improving cardiac function and inhibiting apoptosis. However, the specific mechanisms underlying its efficacy are mostly unknown. This study aimed to evaluate whether LQF could alleviate cardiac injury and apoptosis by regulating the NLRP3 inflammasome and the caspase-3/Bax pathway.

**Purpose:**

In this study, we investigated the effects of LQF on cardiac remodeling in a mouse model of myocardial infarction (MI) in vivo.

**Methods:**

Forty male C57BL/6 mice were randomly divided into four groups: the sham group, the model group, the LQF group, and the perindopril group, with a sample size (*n*) of 10 mice in each group. Except the sham group, the other groups received left anterior descending (LAD) coronary artery ligation to induce MI and then treated with LQF, perindopril, or saline. Six weeks after MI, echocardiography was used to evaluate cardiac structure and function. Myocardial tissue morphology was observed by haematoxylin and eosin (H&E) staining, and heart samples were stained with Masson's trichrome to analyse myocardial fibrosis. Myocardial hypertrophy was observed by fluorescent wheat germ agglutinin (WGA) staining. The expressions of NLRP3, ASC, Cle-caspase-1, IL-1*β*, TXNIP, Cle-caspase-3, Bcl-2, and Bax in heart tissues were assessed by western blot analysis. mRNA expressions of ANP and BNP in heart tissues were measured by RT-PCR. The expression of reactive oxygen species in myocardial tissue was detected by using a DCFH-DA probe.

**Results:**

Echocardiographic analysis showed that compared with the model group, the left ventricular ejection fraction (LVEF) and left ventricular fractional shortening (LVFS) in the LQF and perindopril group were increased (*P* < 0.05), left ventricular internal diameter end diastole (LVIDd) and left ventricular internal diameter end-systole (LVIDs) were reduced (*P* < 0.05), and H&E and Masson's trichrome staining of cardiac tissues showed that LQF and perindopril could partially reverse ventricular remodeling and alleviate myocardial fibrosis (*P* < 0.05). WGA fluorescence results showed that compared with the model group, myocardial hypertrophy was significantly reduced in the LQF and perindopril group. We also found that LQF and perindopril reduce the oxidative stress response in the heart of MI mice. The protein expression of NLRP3, ASC, Cle-caspase-1, IL-1*β*, TXNIP, Cle-caspase-3, and Bax was downregulated in the LHF and perindopril treatment group, and Bcl-2 expression was upregulated.

**Conclusion:**

LQF and perindopril significantly attenuated cardiac injury and apoptosis in the MI model. In addition, we found that LQF effectively inhibited the activation of the NLRP3/ASC/caspase-1/IL-1*β* cascade, decreased inflammatory infiltration, delayed ventricular remodeling, and downregulated caspase-3/Bax signaling, which can effectively reduce the apoptosis of cardiomyocytes. Perindopril showed the same mechanism.

## 1. Introduction

Chronic heart failure (CHF), characterized by high mortality and morbidity, is the final outcome of many CVDs [1]. Myocardial infarction (MI) is one of the most important causes of heart failure (HF), and how to reduce cardiac dysfunction and HF risk after MI has always been the focus of CVD research [2].

Growing evidence suggests that an aseptic inflammatory response during acute myocardial ischemia (AMI) plays a crucial role in the formation of scar and remodeling of the damaged myocardium [3]. An early inflammatory response might increase matrix degradation, causing the infarcted myocardial tissue to heal and form a scar. However, the aggravation, prolongation, or extension of the inflammatory response might lead to the deterioration of the remodeling and dysfunction after MI [4]. Therefore, moderate regulation of the inflammatory response after MI is very important for myocardial remodeling of the left ventricle to prevent HF [5]. Targeted inhibition of most of the induced inflammatory responses could be a potential treatment for MI.

Many studies have found that activation of the NLRP3 protein leads to an enhanced inflammatory response and cardiac dysfunction and remodeling, which aggravates acute myocardial injury in MI animals [6, 7]. In contrast, a lack of NLRP3 inflammasome components might reduce the inflammatory response and promote cardioprotective effect [8]. The NLRP3 inflammasome is a supramolecular protein complex that activates caspase-1 and modulates the maturation of interleukin 1*β* (IL-1*β*), thereby initiating the inflammatory response [9]. Recent research has shown that NLRP3 plays an important role in CVDs [10]. Gao et al. found that targeting the NLRP3 inflammasome can effectively reduce myocardial fibrosis and cardiac remodeling after MI [11]. Wang et al.'s study has shown that NLRP3 inflammasome is strongly associated with CVB3-induced myocarditis, and in the first 7 days after CVB3 infection, the level of IL-1*β*, cleaved caspase-1, and ASC was increased [12]. Therefore, targeted inhibition of NLRP3 activation could reduce the inflammatory response after MI and delay ventricular remodeling.

Traditional Chinese medicine (TCM) has been widely used in the prevention and treatment of diseases over the years and has been clinically validated as a good means to promote health. In China, it has been well recognized that TCM is markedly effective to treat CHF. LQF, an effective prescription to treat CHF, has been used widely in clinical practice; LQF (Chinese national patent number ZL2014102933164) is an empirical compound prescription of several common medicinal herbs in TCM, composed of antler, safflower, *Astragalus membranaceus*, *Codonopsis pilosula*, *Cassia twig, and*, *Semen lepidii*, aiming at “warming kidney and Eliminating Dampness.” LQF can effectively relieve chest tightness, edema, and other uncomfortable symptoms of CHF patients. However, its effect on ventricular remodeling remains to be studied. As the cornerstone of clinical treatment of HF, angiotensin-converting enzyme inhibitors (ACEIs) improve ventricular remodeling [13, 14]. However, whether ACEIs improve ventricular remodeling in mice with HF in association with downregulation of NLRP3 inflammation remains unclear. Therefore, this study aimed to assess the effects of LQF and the long-acting ACEI perindopril on ventricular remodeling in mice with HF.

## 2. Materials and Methods

### 2.1. Animals and Drugs

Male wild-type (WT) C57BL/6J mice (body weight 20–23 g), aged 8 weeks, were obtained from the Experimental Animal Center (*n* = 10 for each group), Shanghai University of Chinese Medicine. All the animal experimentation was complied with the ARRIVE guidelines, and this study was conducted in accordance with safe animal testing specifications (Safety Certificate Number: SYXK-HU-2020-0009 and Animal Ethics Code PZSHUTCM200807012). All mice were maintained under a controlled temperature (20 ± 5°C) and humidity (55 ± 15%) with a 12 h light/12 h dark cycle. LQF dry powder, made by Shanghai Chinese Traditional Pharmaceutical Technology Co., Ltd. (China), was dissolved in distilled water to produce the equivalent of the crude drug concentration of 1.78 g/kg and was administered intragastrically. The gavage dose (1.65 g/ml) in the present study was determined according to the equivalent patient dose. Perindopril tablets (4 mg/tablet) were obtained from Servier Pharmaceutical Co., Ltd. (Tianjin, China) and used as a positive control. Perindopril tablets were crushed and dissolved in double-distilled water at a concentration of 0.06 mg/ml. The sham group mice received water. The starting dose for all mice was from the day after LAD artery ligation, and all mice were treated for 6 weeks.

### 2.2. Mouse Model of MI

MI surgery was performed as described previously [15]. The mice were anesthetized and mechanically ventilated using a rodent ventilator. Their hearts were exposed by thoracotomy and subsequent pericardiotomy. MI was induced by permanent LAD coronary artery ligation using 7–0 silk suture, and successful induction of AMI was observed when the anterior and apical left ventricular myocardium gradually attained a dull, pale colour with reduced pulse and then confirmed by ST segment elevation on an electrocardiogram. The sham-operated group underwent the same procedure without ligation of the LAD coronary artery.

### 2.3. Ultrasound Echocardiography

Ultrasound echocardiography was performed by using a Vevo 2100 Imaging System (Visual Sonics Inc., Toronto, ON, Canada) in mice under anesthesia with isoflurane (RWD Life Science Co., Guangdong, China). Briefly, the mice were anesthetized using 1.0–2.5% isoflurane, and the heart rate was maintained at ∼450 to 550 beats/min. The heart was examined in the long-axis view at the papillary muscle level, and an M-mode echocardiogram of the mid ventricle was recorded. Cardiac function indices including LVEF, LVFS, LVIDd, and LVIDs were recorded [16].

### 2.4. Western Blotting

Proteins were isolated from the frozen cardiac tissue, and their concentration was calculated using the bicinchoninic acid (BCA) method (Thermo Fisher Scientific, Waltham, MA, USA; A53225). Immunoblotting was performed as previously described [15]. In this study, the primary antibodies used recognized NLRP3(1 : 1000, CST, 15101S), Cle-caspase-1 (1 : 1000, Abcam, ab1789515), ASC (1 : 1000, CST, 67824), thioredoxin-interacting protein (TXNIP) (1 : 1000, CST, 14715), caspase-3 (1 : 1000, CST, 9662), BCL-2 apoptosis regulator (Bcl-2) (1 : 1000, CST, 3498), IL-1*β* (1 : 1000, Abcam, ab234437), Bax(1 : 1000, Abcam, ab32503), and glyceraldehyde-3-phosphate dehydrogenase (GAPDH) (1 : 20000, Proteintech, 60004-1-Ig). Protein bands were visualized using the chemiluminescence system (Tanon Science & Technology Co., Shanghai, China) for the required time. Quantitative analysis was performed using Image J software (NIH, Bethesda, MD, USA).

### 2.5. Histological Staining and Evaluation

Heart tissues were isolated, rinsed with phosphate buffered saline (PBS), and fixed in 4% paraformaldehyde (PFA) over 24 h. Then, the tissues were dehydrated and paraffin-embedded. Next, 5 *μ*m-thick slices were cut for H&E staining to explore changes in heart size and for Masson's trichrome staining to visualize fibrosis [17]. After staining, all slices were completely scanned using Caseviewer 2.0 (Pannoramic 250/MIDI, 3DHISTECH, Budapest, Hungary). The severity of cardiac fibrosis was calculated by the Masson's trichrome-positive areas in 4 randomly selected fields per section of cardiac using Image J software.

### 2.6. Immunohistochemical (IHC) and Immunofluorescence (IF) Analysis

Heart tissues were isolated, rinsed with PBS, and fixed in 4% PFA over 24 h. The hearts were then dehydrated and paraffin-embedded. Next, 5 *μ*m-thick slices were cut for IHC and IF analyses. Paraffin sections were dewaxed with water, and antigens were retrieved by heating in sodium citrate buffer. Endogenous peroxidase was removed by adding 30% H_2_O_2_, and an immunohistochemical pen was used to draw a circle around the tissue. Subsequently, 10% fetal bovine serum was added to block nonspecific binding to the tissue. The sections were incubated with NLRP3 and anti-IL-1*β* primary antibodies overnight at 4°C. Goat-anti-mouse secondary antibodies were then added and incubated at room temperature for 60 min, followed by washing with PBS three times for 5 min. Then, 30 *μ*L of 4′, 6-diamidino-2-phenylindole (DAPI) solution was added and incubated for 10 min, followed by three PBS washes. For immunofluorescence staining, WGA(GeneTex, USA, GTX01502) was diluted to 0.1 mg/mL, added to the sections, and incubated at room temperature for 2 h. Sections were then washed three times with PBS for 5 min. Then, 30 *μ*L of DAPI solution was added to the section and incubated for 10 min, followed by three PBS washes. A fluorescence microscope was used to observe and collect images in a darkroom. Semiquantitative analysis was performed in four randomly selected fields per section using Image J software.

### 2.7. Terminal Deoxynulceotidyl Transferase Nick-End-Labeling (TUNEL) Assay

Paraffin-embedded cardiac tissues were sectioned into 5 *μ*m slices. After dewaxing to water, 20 *μ*g/mL proteinase K was added to cover the tissue. Twenty minutes later, the sections were washed with PBS three times for 5 min, and TUNEL dye (C1086, Beyotime Biotechnology, Shanghai, China) was added and incubated at 37°C for 1 h. The slices were then washed with PBS three times for 5 min. DAPI was added to slices and incubated for 10 min. The slices were sealed with an antifluorescence quenching sealant. Fluorescence microscopy was performed in a darkroom for observation and image collection.

### 2.8. Reactive Oxygen Species (ROS) Analysis

Heart tissue was separated, washed with PBS, and rapidly frozen in liquid nitrogen. The tissue was then embedded with an optimal cutting temperature (OCT) embedding agent. After embedding, 6 *μ*m-thick sections were generated using a freezing microtome for ROS staining. Frozen sections were fixed at room temperature with 4% PFA for 5 minutes and washed three times with PBS for 1 minute each, and then, a tissue pen was used to draw a circle around the tissue. 5 *μ*M 20,70-dichlorofluorescein diacetate (DCFH-DA) was added to the circled area and incubated at 37°C for 20 min, avoiding light, followed by PBS washes. DAPI staining was carried out for 10 min in the dark at room temperature. Slices were then washed with PBS three times for 5 min each. The slices were sealed with an antifluorescence quenching sealant. Fluorescence microscopy was performed in a darkroom for observation and image collection.

### 2.9. Malondialdehyde (MDA), Superoxide Dismutase (SOD), and Glutathione (GSH) Analysis

The contents of MDA (A003-1-2, Jiancheng Bioengineering Institute, Nanjing, China), SOD (A001-3-2, Jiancheng Bioengineering Institute), and GSH (A005-1-2, Jiancheng Bioengineering Institute) were determined by colorimetry. The assays were performed according to the manufacturer's recommendations.

### 2.10. RNA Extraction and RT-PCR

Total RNA was isolated from individual hearts using commercial kits to evaluate mRNA expression of atrial natriuretic peptide (ANP) and brain type natriuretic peptide (BNP). Reverse-transcribed cDNA was obtained using the Total RNA rapid extraction kit (BioTeke, China, RP4002). The primers used to amplify the genes are listed in Table 1. All primer sequences were checked in GenBank to avoid inadvertent sequence homologies. They were designed and synthesized by Sangon Biotechnology (Shanghai, China). Reactions were performed using an SYBR Green PCR master mix (Vazyme, China, Q711-02). Relative amounts of mRNA for specific genes were calculated using the 2−^ΔΔ^Ct values [18].

### 2.11. Statistical Analysis

All results are expressed as the mean ± standard deviation (SD) and were analyzed using one-way analysis of variance (ANOVA) with the least significant difference- (LSD-) t's multiple comparisons, using SPSS version 20.0 software (IBM Corp., Armonk, NY, USA). *P* < 0.05 was considered statistically significant.

## 3. Results

### 3.1. LQF Improved Cardiac Function after MI

**Figure 1:**
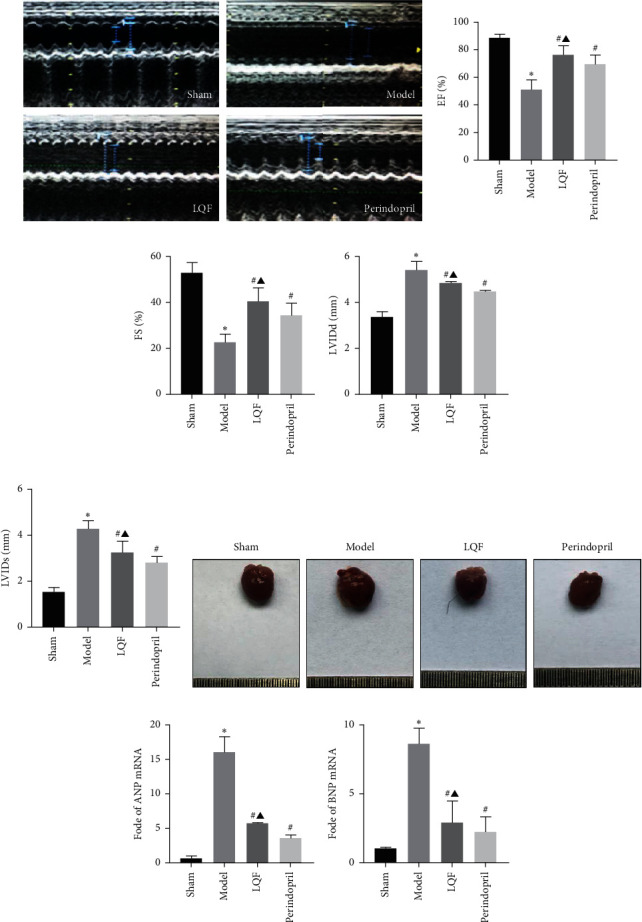
LQF improves cardiac function after MI. (a) Representative images of echocardiography tracings of the mice 6 weeks after MI in four groups. (b) EF%, (c) FS%, (d) LVIDd, and (e) LVIDs. (f) Representative gross morphology of whole hearts. (g), (h) Transcript levels of *Anp* and *Bnp*, as determined by qRT-PCR. ^*∗*^*P* < 0.05*vs.* the sham group, ^#^*P* < 0.05*vs.* the model group, and ^▲^*P* < 0.05*vs.* the perindopril group.

Cardiac ultrasound is an important method to evaluate cardiac function, and MI results in poor ventricular remodeling [19]. As shown in Figures 1(a)–1(f), in the MI model group, the ejection fraction (EF) and fractional shortening (FS) values were reduced, and LVIDd and LVIDs were augmented compared with the sham group, whereas LQF and perindopril treatment reduced the deterioration of cardiac function and cardiac enlargement. *ANP* and *BNP* are sensitive markers of myocardial dysfunction, which correlate positively with the severity of HF. Thus, ANP and BNP are important indicators to determine the progression and prognosis of HF [20]. The qRT-PCR analysis showed that MI-induced increases of *Anp* and *Bnp* mRNA expression were partly alleviated in LQF and perindopril mice compared with model mice (Figures 1(g) and 1(h)). This revealed that LQF and perindopril have protective effects against cardiac function. No significant difference in the cardiac function between the LQF group and the perindopril group was noted.

ACE inhibitor treatment improves morbidity and mortality in post-MI patients [21], and related studies have shown that perindopril can delay myocardial remodeling and myocardial fibrosis. Raj et al. found that resveratrol and perindopril can attenuate cardiac remodeling and contractile dysfunction in rats post infarct [22]. Li et al. have demonstrated that perindopril downregulates Gal-3 and reduces myocardial fibrosis [23]. The intragastric doses of perindopril and LQF in this experiment were both within the safe range, so no obvious adverse reactions occurred during the experiment.

### 3.2. LQF Reduced Myocardial Fibrosis and Hypertrophy

**Figure 2:**
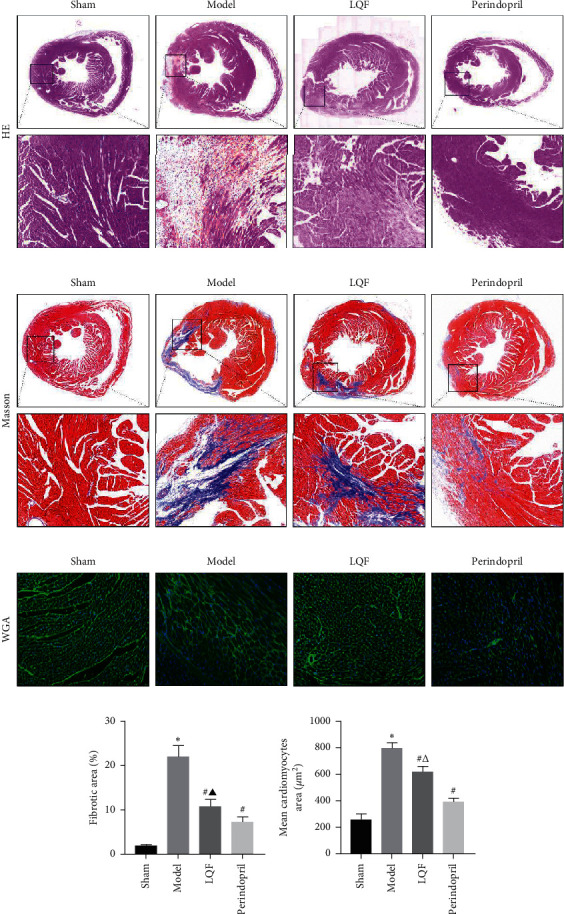
LQF reduces myocardial fibrosis and hypertrophy. (a) Representative image of H&E staining showing the size of mouse myocytes (the scale bar of the picture above is 1 mm, and the scale bar of the picture below is 100 *μ*m) (*n* = 3). (b) Representative Masson's trichrome staining of the heart sections (the scale of the picture above is 1 mm, and the scale of the picture below is 100 *μ*m) (*n* = 3). (c) Representative images of WGA staining of heart sections (magnification 200×) (*n* = 3). (d) Quantitative analysis of the fibrotic area. (e) Mean cardiomyocytes area. ^*∗*^*P* < 0.05*vs.* the sham group, ^#^*P* < 0.05*vs.* the model group, ^▲^*P* < 0.05*vs.* the perindopril group, and ^△^*P* < 0.05*vs.* the perindopril group.

Interstitial fibrosis is the hallmark of cardiac remodeling [24]. To determine whether LQF and perindopril could ameliorate cardiac fibrosis and hypertrophy after MI, we analyzed myocardial injury and fibrosis by H&E and Masson staining. In the sham group, the myocardial fibers were arranged regularly, and there was no exudation, edema, or inflammatory cell infiltration in the myocardial interstitium. No abnormal, blue-stained collagen fibers were found in the myocardial interstitium. In the model group, there were degeneration and edema of cardiomyocytes, uneven staining of myocardial fibers, infiltration of inflammatory cells in the epicardium, staggered distribution of blue-stained collagen fibers, and increased distribution of myocardial fibers. In the LQF group, the myocardial injury was alleviated, the infiltration of inflammatory cells was significantly reduced, and a small amount of blue-stained collagen fibers could be seen. The myocardial morphology of the perindopril group was similar to that of the LQF group (Figures 2(a) and 2(b)).

WGA is an effective indicator of cardiac hypertrophy [25]. As shown in Figures 2(c) and 2(e), compared with the sham group, the cardiomyocytes in the model group were enlarged, the myocardial fiber outline was irregular and deformed, and the number of cells was decreased. Compared with the model group, the volume of cardiomyocytes in LQF and perindopril groups was smaller, the outlines of the myocardial fibers were relatively regular, and the number of cardiomyocytes was increased. Furthermore, the effect of perindopril on reducing cardiomyocyte hypertrophy was more significant.

### 3.3. LQF Inhibited NLRP3 Inflammasome Activation

**Figure 3:**
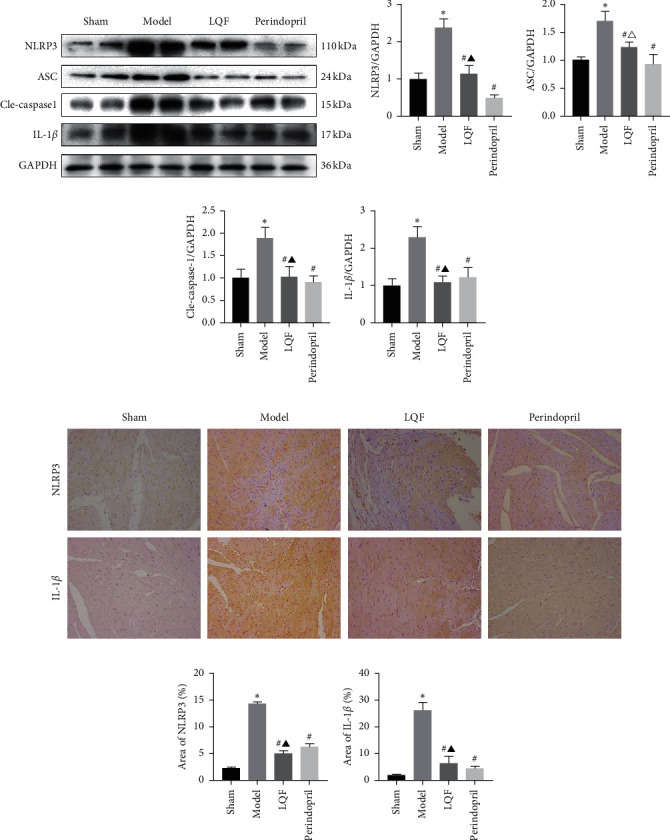
LQF inhibits NLRP3 inflammasome activation. (a)–(e) Representative western blotting and quantification of inflammation-related proteins, including NLRP3, ASC, Cle-caspase-1, and IL-1*β*, GAPDH served as a loading control (*n* = 5). (f)–(h) Representative image of a cardiac section stained for NLRP3 and IL-1*β*, with the quantification results of IHC (magnification 200×) (*n* = 3). ^*∗*^*P* < 0.05*vs.* the sham group, ^#^*P* < 0.05*vs.* the model group, ^▲^*P* < 0.05*vs.* the perindopril group, and ^△^*P* < 0.05*vs.* the perindopril group.

An aseptic inflammatory response during MI plays an important role in scar formation and myocardial remodeling. Moderate regulation of the inflammatory response after MI is very important to prevent HF; however, an excessive inflammatory response will lead to the deterioration of myocardial remodeling and dysfunction after MI. Many studies have found that NLRP3 is activated after MI, resulting in an enhanced inflammatory response and cardiac dysfunction [26]. To determine whether the effect of LQF and perindopril on improving cardiac function is related to the inflammasome, we studied the expression of NLRP3 inflammasome in the heart. Unsurprisingly, NLRP3, ASC, Cle-caspase-1, and IL-1*β* protein levels in the cardiac tissue of the mice in the model group were significantly higher than those in the other groups. After treating with LQF and perindopril, mice after MI had reduced levels of NLRP3, ASC, Cle-caspase-1, and IL-1*β* (Figures 3(a)–3(e)). In addition, immunohistochemical staining further showed that LQF and perindopril could reduce the levels of NLRP3 and IL-1*β* after MI (Figures 3(f)–3(h)). Moreover, compared with perindopril, LQF showed no significant difference in inhibiting the NLRP3 and IL-1*β* after MI. These findings indicated that LQF and perindopril can effectively reduce the levels of proteins associated with the NLRP3 inflammasome and cytokine IL-1*β*.

### 3.4. LQF Suppressed the Rise of ROS and Reduced the Oxidative Stress Response in the Hearts of MI Mice

**Figure 4:**
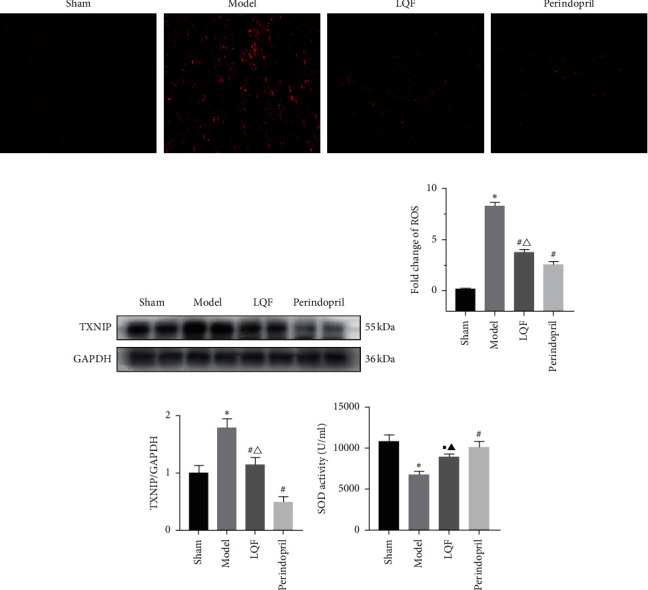
LQF reduces the oxidative stress response in the heart of MI mice. (a) Representative images of ROS in a section of mouse heart assessed using immunofluorescence staining (magnification 200×) (*n* = 3). (b) Representative western blotting of TXNIP (*n* = 5). (c)-(d) Quantification of the ROS assay and TXNIP. (e)–(g) SOD, MDA, and GSH levels in whole ventricular lysates, as measured using a colorimetric method, ^*∗*^*P* < 0.05 vs. the sham group, ^#^*P* < 0.05 vs. the model group, ^▪^*P* < 0.05 vs. the model group, ^△^*P* < 0.05 vs. the perindopril group, and ^▲^*P* < 0.05 vs. the perindopril group.

After MI, the necrotic cells and matrix fragments in the infarct area produce damage-associated molecular patterns (DAMPs) signaling molecules, leading to the increase of mitochondrial ROS and activation of NLRP3 inflammasome. To clarify whether the inhibition of the NLRP3 inflammasome by LQF and perindopril is related to the inhibition of ROS, we detected the ROS levels in the cardiac tissue (Figures 4(a) and 4(c)). The ROS staining results showed that a higher level of ROS was observed in the model group. However, LQF and perindopril reduced the production of ROS. By contrast, ROS could powerfully trigger the association of TXNIP with NLRP3. As shown in Figure 4(b), TXNIP levels in the model group were significantly higher than those in the other groups. In the LQF and perindopril groups, TXNIP levels were particularly suppressed (Figure 4(d)). Meanwhile, we assessed levels of SOD, MDA, and GSH (Figures 4(e)–4(g)), which are all closely related to oxidative stress (OS). The results showed that compared with the sham mice, the level of MDA was significantly increased, while the levels of SOD and GSH were significantly decreased in the model group. Compared with those in the model mice, the level of MDA was significantly decreased, and the levels of SOD and GSH were increased in the LQF group. In addition, SOD and GSH levels in the perindopril group increased more significantly. These results suggest that LQF can repair OS-induced cardiac injury.

### 3.5. LQF Inhibited Apoptosis in Mice after MI

**Figure 5:**
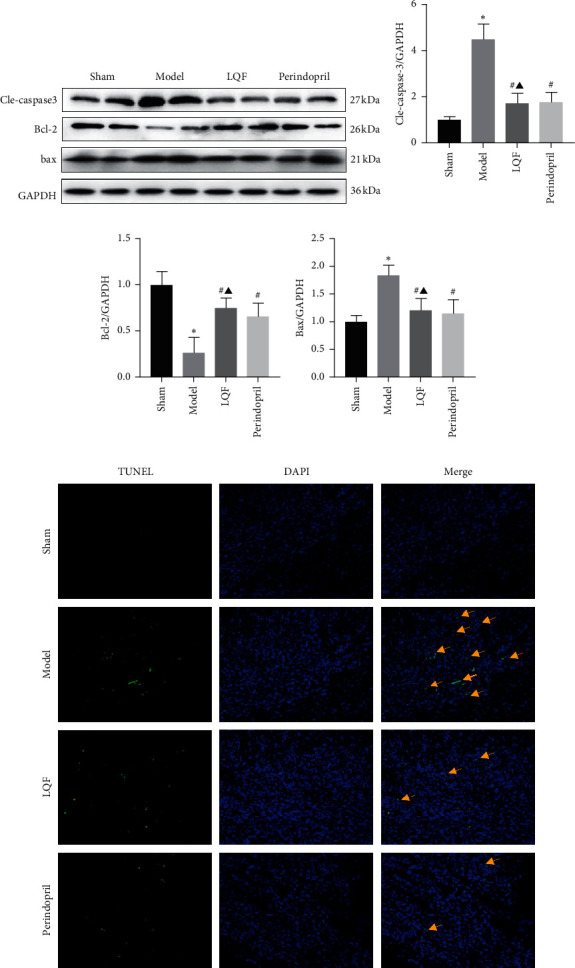
LQF inhibited apoptosis in mice after MI. (a)–(d) Representative western blotting and quantification of apoptosis-related proteins, including Cle-caspase-3, Bcl-2, and Bax, and GAPDH served as a loading control (*n* = 5). ^*∗*^*P* < 0.05*vs.* the sham group, ^#^*P* < 0.05*vs.* the model group, and ^▲^*P* < 0.05*vs.* the perindopril group. (e) TUNEL staining of apoptotic nuclei in cardiac tissues (magnification 200×) (*n* = 3).

To identify whether LQF protects the postinfarct myocardium against apoptosis, we assessed apoptotic biomarker levels using western blotting and TUNEL staining. The results show that there was a significant increase in Bcl-2 and a decrease in Cle-caspase-3 and Bax levels on the border of the MI hearts after LQF and perindopril treatment (Figures 5(a)–5(d)), suggesting that LQF and perindopril have antiapoptotic properties. This result was confirmed by the TUNEL staining. There were dramatically fewer apoptotic (TUNEL-positive) cells on the border of the MI hearts of the LQF and perindopril group compared to that of the model group (Figure 5(e)). According to the results, MI stimulates excess ROS production and activates OS in cardiac tissues, which affects TXNIP activity and prompts TXNIP to bind with NLRP3, further triggering the assembly of the NLRP3 inflammasome. This process continues to activate the NLRP3-ASC-caspase-1 axis to release inflammatory factors and trigger cardiac inflammatory responses, which eventually leads to apoptosis of the cardiomyocytes. LQF suppresses the activation of the NLRP3-ASC-caspase-1 axis by inhibiting TXNIP, thus ameliorating cardiac apoptosis.

## 4. Discussion

Chronic heart disease is associated with a persistent low-grade inflammatory response that promotes adverse cardiac remodeling and is correlated with disease progression [27]. Currently, anti-inflammatory therapies for CVDs, such as those investigated in the Canakinumab Anti-inflammatory Thrombosis Outcomes Study (CANTOS) and Colchicine Cardiovascular Outcomes Trial, have shown effectiveness [28, 29]. This study demonstrated that MI in mice induced cardiac dysfunction and the occurrence of an inflammatory response, especially the activation of the NLRP3 inflammasome. The results showed that LQF exerted potent protection against MI and suppressed inflammation in mice. Our results revealed that LQF significantly inhibited the activation of the NLRP3 inflammasome and could be attributable to suppressing the ROS-TXNIP-NLRP3 pathway.

As a macromolecular complex, the NLRP3 inflammasome has been reported to be involved in many CVDs, including atherosclerosis, ischemia/reperfusion (I/R) injury, and HF [30]. A growing body of evidence has revealed that the NLRP3 inflammasome plays a critical role in promoting sterile inflammation through the recognition of danger signals released from dying cells [31]. Therefore, inhibiting the NLRP3 inflammasome is expected to be a novel approach to treating HF [10]. After MI, necrotic cells and stromal debris in the infarcted area produce DAMPs that bind to pattern recognition receptors (PRRs), activate the innate immune system, and produce a strong inflammatory response, leading to an increase in mitochondrial ROS and activation of the NLRP3 inflammasome. Sandanger et al. have found that NLRP3-deficient mice subjected to myocardial I/R injury had a smaller infarct size and preserved cardiac function compared with those of WT mice [32]. Moreover, many active ingredients of LQF, such as carthamin yellow from safflower, astragaloside IV from *Astragalus membranaceus*, and atractylodesin III from *Codonopsis pilosula*, have shown anti-inflammatory effects in CVDs [33–35]. In this study, we found that LQF had a significant inhibitory effect on the activation of NLRP3 inflammasomes in MI mice by inhibiting the secretion of IL-1*β* and downregulating the activity of caspase-1. The results suggested that LQF can be potentially developed as NLRP3-targeting medicine against MI-associated diseases.

The common upstream mechanisms of the NLRP3 inflammasome activation are believed to include intracellular K^+^ outflow, lysosomal breakdown, and production of ROS. Mitochondrial dysfunction leads to changes in the ROS concentration, and the thioredoxin interacting protein separates from the thioredoxin and binds to NLRP3, inducing activation of the NLRP3 inflammasome [36]. The findings of numerous previous studies have verified that ROS activates the NLRP3 inflammasome through TXNIP, triggering a rapid cardiac inflammation response [37–39]. In the present study, we observed high expression of mitochondrial ROS and TXNIP in cardiac tissue, which triggered NLRP3 overexpression and cardiac inflammation. However, ROS was suppressed by LQF intervention, which in turn suppressed OS and maintained the TXNIP-Trx bond. This bond suppressed TXNIP activity and prevented it from binding with NLRP3, thereby preventing the activation of the inflammasome axis and ameliorating cardiac inflammation and injury. After MI, inflammatory reactions are triggered by DAMPs, which activate macrophages to coordinate inflammation and tissue repair, however, an excessive inflammatory response leads to myocardial apoptosis [40]. Therefore, we examined the apoptosis of cardiomyocytes. Our data showed that Cle-caspase-3 and Bax levels were upregulated in the model group. Conversely, Bcl-2 levels were upregulated significantly after treatment with LQF. The TUNEL assay results also proved the efficacy of LQF against myocardial apoptosis, verifying that one of the mechanisms by which LQF attenuates cardiac apoptosis could be to inhibit the inflammatory response induced by activation of the NLRP3 inflammasome.

In summary, our study demonstrates that LQF prevents ventricular remodeling after MI by regulating NLRP3 inflammasome. The application of Chinese medicine reflects advantages of simplicity and efficiency. Therefore, LQF should be used as a carrier to explore the mechanism of improving ventricular remodeling after MI.

## 5. Limitations

In this study, we only verified the effect of LQF on inhibiting the NLRP3 inflammasome and reducing myocardial apoptosis in animal experiments; therefore, which active component in LQF exerts these specific effects needs to be further clarified in our subsequent studies.

## 6. Conclusions

In summary, we verified that, by inhibiting the production of ROS in cardiac tissue, LQF inhibits NLRP3/ASC/caspase-1/IL-1*β* pathway activation, thereby alleviating cardiac inflammation, fibrosis, and apoptosis. We found that LQF demonstrated excellent performance in protecting cardiac function and ameliorating cardiac inflammation and apoptosis. Interestingly, we also found that perindopril reduced cardiomyocyte apoptosis by inhibiting NLRP3 inflammasome. The present study delineated the therapeutic properties of LQF and its value for subsequent research and clinical applications.

## Figures and Tables

**Table 1:** PCR primer sequence.

Gene name	Primer sequence (5′–3′)	Tm (°C)
*Anp*	F5′GCTTCCAGGCCATATTGGAG3′	**60.2**
	R5′GGGGGCATGACCTCATCTT3′	**61.1**

*Bnp*	F5′GAGGTCACTCCTATCCTCTGG3′	**60.2**
	R5′GCCATTTCCTCCGACTTTTCTC3′	**61.2**

## Data Availability

The data used to support the findings of this study are available from the corresponding author upon request.
